# Increasing Ceftriaxone Resistance in Salmonellae, Taiwan

**DOI:** 10.3201/eid1706.101949

**Published:** 2011-06

**Authors:** Lin-Hui Su, Wen-Shin Teng, Chyi-Liang Chen, Hao-Yuan Lee, Hsin-Chieh Li, Tsu-Lan Wu, Cheng-Hsun Chiu

**Affiliations:** Author affiliations: Chang Gung Memorial Hospital, Taoyuan, Taiwan (L.-H. Su, H.-C. Li, T.-L. Wu);; Chang Gung University College of Medicine, Taoyuan (L.-H. Su, W.-S. Teng, H.-Y. Lee, T.-L. Wu, C.-H. Chiu);; Chang Gung Children’s Hospital, Taoyuan (W.-S. Teng, C.-L. Chen, H.-Y. Lee, C.-H. Chiu)

**Keywords:** Salmonella enterica serotype Choleraesuis, nontyphoidal Salmonella, antimicrobial resistance, salmonellae, CMY-2, Tn6092, conjugative resistance plasmid, bacteria, Taiwan, letter

## Abstract

In Taiwan, despite a substantial decline of *Salmonella enterica* serotype Choleraesuis infections, strains resistant to ciprofloxacin and ceftriaxone persist. A self-transferable *bla*_CMY-2_-harboring IncI1 plasmid was identified in *S. enterica* serotypes Choleraesuis, Typhimurium, Agona, and Enteritidis and contributed to the overall increase of ceftriaxone resistance in salmonellae.

*Salmonella enterica* serotype Choleraesuis usually causes invasive infection (*1*). When resistant *Salmonella* infection is encountered, fluoroquinolones or extended-spectrum cephalosporins are frequently used (*2*). Fluoroquinolone resistance has been common in this invasive serotype (*3*). Isolation of SC-B67, a strain of *S. enterica* ser. Choleraesuis that was resistant to ciprofloxacin and ceftriaxone (CIP^r^/CRO^r^), has exacerbated the problem (*4*). Ceftriaxone resistance in SC-B67 was attributed to a plasmid-mediated *bla*_CMY-2_, located on a specific IS*Ecp1*-*bla*_CMY-2_-*blc*-*sugE* structure (*4*). This conserved DNA fragment, subsequently named Tn*6092* (*5*), has been reported from different geographic areas and is widely distributed among various *Salmonella* serotypes and other Enterobacteriaceae (*6*).

## The Study

**Figure 1:**
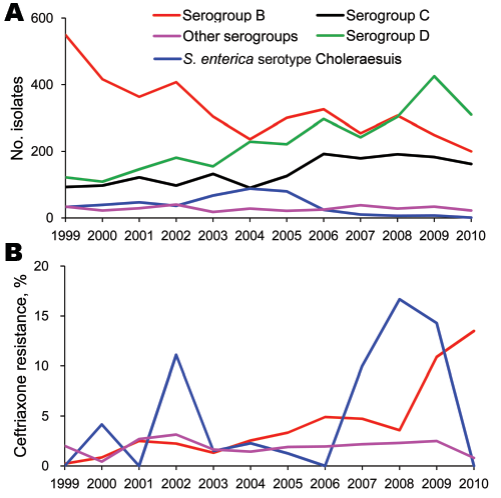
Secular trends in annual numbers (A) and rates (B) of ceftriaxone resistance among various serogroups or serotype of nontyphoidal *Salmonella enterica* isolates in Chang Gung Memorial Hospital, 1999–2010.

Since 1999, computerized records of bacterial culture results have been stored at Chang Gung Memorial Hospital, a 3,500-bed medical center in northern Taiwan. Periodic review of these records indicated a reverse trend in the prevalence of serogroups D (increase) and B (decrease) isolates during the past decade (Figure 1, panel A). A significant decrease in the prevalence of *S. enterica* ser. Choleraesuis was also evident (Figure 1, panel A). Nevertheless, in recent years, ceftriaxone resistance has increased from <5% to >10% in *S. enterica* ser. Choleraesuis and in serogroup B salmonellae (Figure 1, panel B).

Since isolation of SC-B67 in 2002 (*4*), 10 CIP^r^/CRO^r^ *S. enterica* ser. Choleraesuis isolates have been recovered. All CIP^r^/CRO^r^ isolates were resistant to nalidixic acid and ciprofloxacin, but SC-B134 remained susceptible to ciprofloxacin (Table 1). PCR and sequencing with specific primers (Technical Appendix Table 1) revealed 3 identical amino acid changes in GyrA and ParC among all CIP^r^/CRO^r^ isolates except SC-B134 (Table 1). Reduced fluoroquinolone susceptibility of SC-B134 could be explained by the single amino acid change at codon 87 of GyrA. Amino acid changes were not found in GyrB or ParE.

**Table 1:** Characteristics of the resistant Salmonella enterica isolates from 17 patients at Chang Gung Memorial Hospital, Taiwan*

Serotype and isolate (no. patients)	Year	Type of specimen	Susceptibility profile†	Puls‡	Plasmid profile, kb§	DNA–DNA hybridization¶				PCR sequencing#			
						*spvC*	*rep* FIIA/FIB	Rep_3		*gyrA*			*parC*
										Ser(83)	D(87)		Ser(80)
Choleraesuis													
SC-B67	2002	Blood	R/R/R	C-1-a	50, **138**	50	50	138		F	N		I
SC-B104 (1)	2003	Blood	R/R/R	C-1-b	50, **150**	50	50	150		F	N		I
SC-B93 (2)	2003	Blood	R/R/R	C-1-b	50, **115**	50	50	50,115		F	N		I
SC-B98 (3)	2004	Blood	R/R/R	C-1-c	50, **138**	50	50	138		F	N		I
NA (4)	2004	Pus	R/R/R	NA	NA	NA	NA	NA		NA	NA		NA
SC-B131 (5)	2004	Blood	R/R/R	C-1-d	50, **138**	50	50	138		F	N		I
SC-B132 (6)	2004	Blood	R/R/R	C-1-b	50, **150**	50	50	150		F	N		I
NA (7)	2005	Pus	R/S/R	NA	NA	NA	NA	NA		NA	NA		NA
SC-B134 (8)	2007	Blood	R/S/R†	C-1-e	40, 65, **105**	65	65	40		Ser	N		Ser
SC-B136 (9)	2008	Blood	R/R/R	C-2	115, **138**	115	115	115		F	N		I
NA (10)	2009	Pus	R/S/R	NA	NA	NA	NA	NA		NA	NA		NA
Typhimurium var. Copenhagen SB-5	2010	Feces	R/R/R	B-1-a	7, **125**, 180, 260	Neg	Neg	Neg		Ser	D		Ser
Typhimurium													
SB-28	2010	Urine	R/R/S	B-2	**115**, 210	Neg	Neg	Neg		Ser	D		Ser
SB-151	2010	Feces	S/S/S	B-1-b	**85**	Neg	Neg	Neg		Ser	D		Ser
SB-193	2010	Feces	R/R/S	B-2	**105**, 210	Neg	Neg	Neg		Ser	D		Ser
Agona SB-105	2010	Feces	R/S/S	B-3	**95**	Neg	Neg	Neg		Ser	D		Ser
Enteritidis SD-166	2010	Feces	R/S/S	D-1	45, 60, **95**	60	Neg	Neg		Ser	D		Ser

*Puls, pulsotype; R, resistant; F, phenylalanine; N, asparagine; I, isoleucine; NA, not available; S, susceptible; ser, serine; neg, negative reaction; var., variant; D, aspartic acid. †Antimicrobial drug susceptibility to chloramphenicol, trimethoprim/sulfamethoxazole, and quinolones. Results were the same for the 2 quinolones (nalidixic acid and ciprofloxacin) tested, except that SC-B134 was resistant to nalidixic acid and susceptible to ciprofloxacin. All isolates were resistant to ampicillin and ceftriaxone. ‡Pulsed-field gel electrophoresis; pulsotypes are expressed as serogroup-major type-subtype. §Plasmids harboring the *bla*_CMY-2_-carrying Tn*6092* element are shown in **boldface**. IncI1 plasmids are underlined. Both are evidenced by DNA–DNA hybridization. ¶The size (kb) of the plasmid showing positive results in the respective DNA–DNA hybridization experiments is indicated. Rep_3, replicon of pSC138, the Tn*6092*-containing resistant plasmid of strain SC-B67. #Amino acid changes compared with the quinolone resistance–determining regions of *gyrA* (codons 83 and 87) and *parC* (codon 80) in *S*. *enterica* serotype Typhimurium LT2. No mutation was found in *gyrB* and *parE*.

In terms of clinical features (Technical Appendix Table 2), most patients with these infections were adults who had a wide spectrum of underlying diseases. Antimicrobial agents were prescribed for all patients, and extended-spectrum cephalosporins were used most frequently. Two patients died. Seven blood isolates from the 10 patients with CIP^r^/CRO^r^ *S. enterica* ser. Choleraesuis infections, together with the ceftriaxone-resistant isolates noted below, were investigated further. SC-B67 was used as a reference.

**Table 2:** Characteristics of conjugative *bla*_CMY-2_-harboring IncI1 plasmids derived in this study and comparison of pMLST patterns with similar plasmids published previously*

IncI1 plasmid	*Salmonella enterica* serotype or *Escherichia coli*	Susceptibility profile†	Country	Year of isolation	pMLST‡					ST§	Clonal complex	Reference
repI1	*ardA*	*trbA*	*sogS*	*pilL*
pSC-B134	Choleraesuis	R/S/S	Taiwan	2007	1	1	15	9	3	51	NA	This study
pSC-B136	Choleraesuis	R/R/S	Taiwan	2008	1	4	15	11	2	52	NA	This study
pSB-5	Typhimurium variant Copenhagen	R/R/R	Taiwan	2010	2	1	15	11	2	53	NA	This study
pSB28	Typhimurium	S/S/S	Taiwan	2010	1	4	5	11	2	54	NA	This study
pSB151	Typhimurium	S/S/S	Taiwan	2010	4	5	15	11	3	55	NA	This study
pSB193	Typhimurium	S/S/S	Taiwan	2010	1	4	5	11	2	54	NA	This study
pSB105	Agona	R/S/S	Taiwan	2010	2	1	15	11	3	56	NA	This study
pSD166	Enteritidis	R/S/S	Taiwan	2010	2	1	15	11	3	56	NA	This study
398T	*E. coli*	NA	Italy	2006	1	2	3	2	1	2	CC-2	(*12*)
05–1909	Heidelberg	NA	Canada	2005	1	2	3	2	1	2	CC-2	(*13*)
1358T	Thompson	NA	USA	1996	1	3	3	4	1	4	CC-12	(*12*)
DH-20406	Heidelberg	NA	USA	2004	1	4	3	4	1	12	CC-12	(*14*)
06–3048	4,5,12:i:–	NA	Canada	2006	1	4	3	4	1	12	CC-12	(*13*)
06–3539	Agona	NA	Canada	2006	1	4	3	4	1	12	CC-12	(*13*)
N07–0084	*E. coli*	NA	Canada	2005	1	4	3	4	1	12	CC-12	(*13*)
05–2835	Heidelberg	NA	Canada	2005	1	4	3	4	1	12	CC-12	(*13*)
06–3985	Litchfield	NA	Canada	2006	1	4	3	4	1	12	CC-12	(*13*)
05–5567	Typhimurium	NA	Canada	2005	1	4	3	4	1	12	CC-12	(*13*)
N06–523	*E. coli*	NA	Canada	2006	1	4	3	2	1	18	CC-2/ CC-12	(*13*)
N06–0537	*E. coli*	NA	Canada	2006	1	3	3	4	3	19	CC-12	(*13*)
05–6117	4,5,12:1:–	NA	Canada	2006	1	1	3	9	1	20	NA	(*13*)
N07–0093	*E. coli*	NA	Canada	2005	1	1	3	9	1	20	NA	(*13*)
06–0753	Heidelberg	NA	Canada	2006	1	2	11	3	3	21	CC-5	(*13*)
N07–0079	*E. coli*	NA	Canada	2005	1	6	3	4	1	22	CC-12	(*13*)
N07–0081	*E. coli*	NA	Canada	2005	1	2	3	1	1	23	CC-2	(*13*)

*pMLST, plasmid multilocus sequence typing; ST, sequence type; R, resistant; S, susceptible; NA, not applicable. †Antimicrobial drug susceptibility to chloramphenicol, trimethoprim/sulfamethoxazole, and quinolones (nalidixic acid and ciprofloxacin). The transconjugants were also resistant to ampicillin and ceftriaxone. ‡pMLST results were compared to those published in the plasmid MLST website (http://pubmlst.org/plasmid). §STs were designated according to the different combinations of allele variants observed among the IncI1 plasmids.

During the first 6 months of 2010, a total of 6 cases of ceftriaxone-resistant *Salmonella* infection were found: serogroup B in 5 patients (*S*. *enterica* ser. Agona, n = 1; *S*. *enterica* ser. Typhimurium, n = 4, including 1 Copenhagen variant) and serogroup D (*S*. *enterica* ser. Enteritidis) in 1 patient (Table 1). All isolates were derived from fecal specimens of patients <3 years of age, except *S*. *enterica* ser. Typhimurium SB-28, which was isolated from the urine of a 77-year-old patient. In contrast to *S. enterica* ser. Choleraesuis, these ceftriaxone-resistant isolates generally remained susceptible to fluoroquinolones (Table 1).

**Figure 2:**
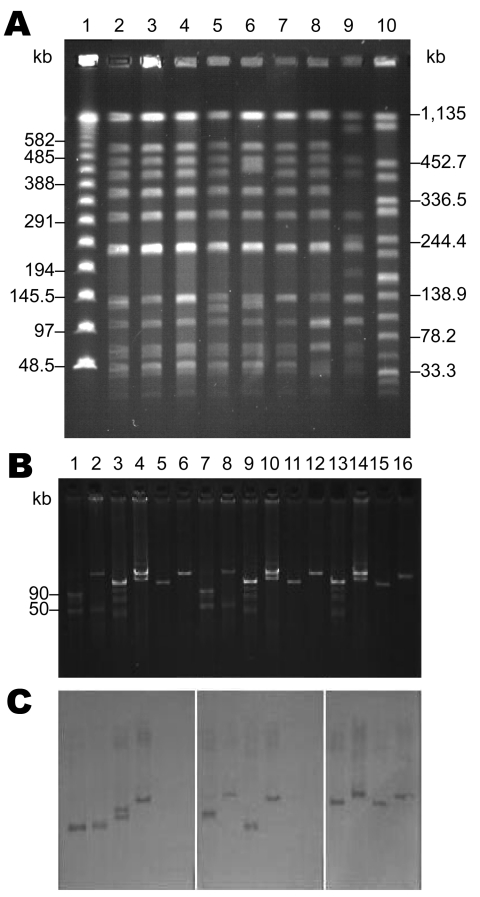
Analyses of *Salmonella* *enterica* serotype Choleraesuis isolates from Chang Gung Memorial Hospital, 1999–2010. A) Pulsed-field gel electrophoresis patterns. Lanes 1 and 10, DNA size markers demonstrated by a λ DNA concatemer standard and *S. enterica* ser. Braenderup H9812, respectively; lanes 2 to 9, *S*. *enterica* ser. Choleraesuis SC-B67, SC-B104, SC-B93, SC-B98, SC-B131, SC-B132, SC-B134, and SC-B136. B) Plasmid analysis and C) DNA–DNA hybridization. Probes for DNA–DNA hybridization of lanes 1–6, 7–12, and 13–16 were prepared from amplicons of *spvC*, Rep_3 replicon of pSC138 in SC-B67, and repI1, respectively; lanes 1 and 7, *S. enterica* ser. Choleraesuis OU7529 containing 2 plasmids of known sizes, 50 kb and 90 kb, was used as the size marker; lanes 2 and 8, SC-B67; lanes 3, 9, and 13, SC-B134; lanes 4, 10, and 14, SC-B136; lanes 5, 11, and 15, *Escherichia coli* J53/pSC-B134; lanes 6, 12, and 16, *E. coli* J53/pSC-B136.

Pulsed-field gel electrophoresis performed as described showed close association among all *S*. *enterica* ser. Choleraesuis isolates, including SC-B67 (Table 1; Figure 2, panel A) (*7*). Only strain SC-B136, recovered in 2008, demonstrated a relatively different pattern. Two pulsotypes, with minor differences, were found among the 4 *S. enterica* ser. Typhimurium isolates (Table 1).

Ceftriaxone resistance was investigated by using PCR and sequencing as described (*6*). The specific *bla*_CMY-2_-carrying Tn*6092* was present in all isolates tested (Table 1). Tn*6092* was located within a *finQ* gene at a position identical to that in SC-B67. The only difference was in SC-B134; a 1,338-bp insertion sequence, IS*10*, was inserted 262 bp upstream of the *bla*_CMY-2_. No CTX-M and SHV genes were found in these isolates.

Using an alkaline lysis method (*8*), we found various numbers of plasmids among the isolates studied (Table 1). DNA–DNA hybridization indicated that Tn*6092* was located on large (85-kb to 150-kb) plasmids (Table 1) (*9*). An identical Rep_3 replicon was found in all CIP^r^/CRO^r^ *S*. *enterica* ser. Choleraesuis isolates studied (*5*). Similar to SC-B67, the Rep_3 replicon was located on the Tn*6092*-harboring resistance plasmid in the 5 resistant isolates recovered before 2004 (Table 1). However, in SC-B134, the Rep_3 replicon was found on the smaller 40-kb plasmid, and in SC-B136, the Rep_3 replicon was on the 115-kb large virulence plasmid that contained the *spvC* gene (Table 1; Figure 2, panels B, C). Replicons FIIA and FIB were simultaneously present in all the *spvC*-containing virulence plasmids among the *S. enterica* ser. Choleraesuis isolates studied (Table 1). Virulence plasmids in the 2 recent isolates, SC-B134 and SC-B136, appeared larger than those in earlier isolates, including SC-B67 (Table 1; Figure 2, panels B, C). The 2 bands demonstrated by the *spvC* probe in SC-B134 were from the same single virulence plasmid, as proven by hybridization experiments on *Bam*HI-digested plasmid DNA of SC-B134 (data not shown).

Replicon typing through a published multiplex PCR system revealed a replicon I1 from the Tn*6092*-harboring resistance plasmids in SC-B134 and SC-B136 (Table 1; Figure 2, panel C) (*10*). Similarly, Tn*6092*-carrying plasmids among the other ceftriaxone-resistant salmonellae isolates all belonged to the IncI1 group (Table 1). Conjugation experiments using a filter mating method showed that all IncI1 resistant plasmids were self-transferrable (*11*). With azide-resistant *Escherichia coli* J53 and *S*. *enterica* ser. Typhimurium LBNP4417 as the recipients, the IncI1-resistant plasmids were confirmed to be self-transferrable.

Subtyping of the 8 conjugated IncI1 plasmids was achieved by using a recently described plasmid multilocus sequence typing (pMLST) method specifically set up for IncI1 plasmids (*12*). Six combinations of allele variants were obtained (Table 2). Because these pMLST patterns differed from those reported elsewhere, 6 new sequence types (STs) were designated (Table 2). Two major groups were further derived: ST54 (pSB28, pSB193) and ST52 (pSC-B136) that differed only in *trbA*, and ST56 (pSD166, PSB105) and ST53 (pSB5) that only differed in *pilL* (Table 2). pMLST patterns of representative *bla*_CMY-2_-carrying IncI1 plasmids published in recent years (Table 2) were derived from *E. coli* or various *Salmonella* serotypes in Europe or North America (*12**–**14*). Nine STs and 2 major clonal complexes, CC-2 and CC-12, were observed. pMLST patterns found in the present study differed from these STs by at least 3 alleles (Table 2).

## Conclusions

Resistance to ciprofloxacin and ceftriaxone remains high, indicating persistence of antimicrobial drug–resistant traits in *S. enterica* ser. Choleraesuis. The conserved genotypes found in the clinical isolates suggest a mode of clonal dissemination. However, plasmid analysis indicates that the location of the Tn*6092*-containing resistance element had shifted from the nonconjugative Rep_3 plasmids in early isolates to the self-transferable IncI1 plasmids in recent isolates. The emergence of such self-transferable resistance plasmids seems to provide an efficient way for *S. enterica* ser. Choleraesuis to spread its ceftriaxone resistance trait.

Because infections with nontyphoid salmonellae are rampant in Asia, emergence of a conjugative IncI1 resistance plasmid in ceftriaxone-resistant salmonellae from an Asian country is of public health concern. Presence of *bla*_CMY-2_-carrying IncI1 plasmids in a variety of *Salmonella* serotypes has been reported, but to our knowledge, not in *S. enterica* serotypes Enteritidis or Choleraesuis (*12**–**14*). IncI1 plasmids of the same or similar STs have been found in isolates of different bacterial species; with different resistance genes; or from different countries or sources, including human, animals, and the environment (*12**–**15*). Emergence of the IncI1 plasmid in Taiwan represents a need for continuous efforts to monitor and control its further spread.

## References

[1] Vugia DJ, Samuel M, Farley MM, Marcus R, Shiferaw B, Shallow S, Invasive *Salmonella* infections in the United States, FoodNet, 1996–1999: incidence, serotype distribution, and outcome. Clin Infect Dis. 2004;38(Suppl 3):S149–56. 10.1086/381581 15095184

[2] Su LH, Chiu CH, Chu C, Ou JT. Antimicrobial resistance in nontyphoid *Salmonella* serotypes: a global challenge. Clin Infect Dis. 2004;39:546–51. 10.1086/422726 15356819

[3] Chiu CH, Wu TL, Su LH, Chu C, Chia JH, Kuo AJ, The emergence in Taiwan of fluoroquinolone resistance in *Salmonella enterica* serotype choleraesuis. N Engl J Med. 2002;346:413–9. 10.1056/NEJMoa012261 11832529

[4] Chiu CH, Su LH, Chu C, Chia JH, Wu TL, Lin TY, Isolation of *Salmonella enterica* serotype choleraesuis resistant to ceftriaxone and ciprofloxacin. Lancet. 2004;363:1285–6. 10.1016/S0140-6736(04)16003-0 15094275

[5] Ye J, Su LH, Chen CL, Hu S, Wang J, Yu J, Complete nucleotide sequence of pSC138, the multidrug resistance plasmid of *Salmonella enterica* serotype Choleraesuis SC-B67. Plasmid. 2011;65:132–40. 10.1016/j.plasmid.2010.11.007 21111756

[6] Su LH, Chen HL, Chia JH, Liu SY, Chu C, Wu TL, Distribution of a transposon-like element carrying *bla*_CMY-2_ among *Salmonella* and other Enterobacteriaceae. J Antimicrob Chemother. 2006;57:424–9. 10.1093/jac/dki478 16396917

[7] Su LH, Leu HS, Chiu YP, Chia JH, Kuo AJ, Sun CF, Molecular investigation of two clusters of nosocomial bacteraemia caused by multiresistant *Klebsiella pneumoniae* using pulsed-field gel electrophoresis and infrequent-restriction-site PCR. J Hosp Infect. 2000;46:110–7. 10.1053/jhin.2000.0815 11049703

[8] Kado CI, Liu ST. Rapid procedure for detection and isolation of large and small plasmids. J Bacteriol. 1981;145:1365–73. 7009583 10.1128/jb.145.3.1365-1373.1981 PMC217141

[9] Southern EM. Detection of specific sequences among DNA fragments separated by gel electrophoresis. J Mol Biol. 1975;98:503–17. 10.1016/S0022-2836(75)80083-0 1195397

[10] Carattoli A, Bertini A, Villa L, Falbo V, Hopkins KL, Threlfall EJ. Identification of plasmids by PCR-based replicon typing. J Microbiol Methods. 2005;63:219–28. 10.1016/j.mimet.2005.03.018 15935499

[11] Jacoby GA, Han P. Detection of extended-spectrum β-lactamases in clinical isolates of *Klebsiella pneumoniae* and *Escherichia coli.* J Clin Microbiol. 1996;34:908–11. 8815106 10.1128/jcm.34.4.908-911.1996 PMC228915

[12] García-Fernández A, Chiaretto G, Bertini A, Villa L, Fortini D, Ricci A, Multilocus sequence typing of IncI1 plasmids carrying extended-spectrum β-lactamases in *Escherichia coli* and *Salmonella* of human and animal origin. J Antimicrob Chemother. 2008;61:1229–33. 10.1093/jac/dkn131 18367460

[13] Mataseje LF, Baudry PJ, Zhanel GG, Morck DW, Read RR, Louie M, Comparison of CMY-2 plasmids isolated from human, animal, and environmental *Escherichia coli* and *Salmonella* spp. from Canada. Diagn Microbiol Infect Dis. 2010;67:387–91. 10.1016/j.diagmicrobio.2010.02.027 20638610

[14] Folster JP, Pecic G, Bolcen S, Theobald L, Hise K, Carattoli A, Characterization of extended-spectrum cephalosporin-resistant *Salmonella enterica* serovar Heidelberg isolated from humans in the United States. Foodborne Pathog Dis. 2010;7:181–7. 10.1089/fpd.2009.0376 19785533

[15] Cloeckaert A, Praud K, Lefevre M, Doublet B, Pardos M, Granier SA, IncI1 plasmid carrying extended-spectrum-β-lactamase gene *bla*_CTX-M-1_ in *Salmonella enterica* isolates from poultry and humans in France, 2003 to 2008. Antimicrob Agents Chemother. 2010;54:4484–6. 10.1128/AAC.00460-10 20643895 PMC2944622

